# Digital innovation in healthcare: quantifying the impact of digital sepsis screening tools on patient outcomes—a multi-site natural experiment

**DOI:** 10.1136/bmjhci-2024-101141

**Published:** 2025-04-27

**Authors:** Kate Honeyford, Alf Timney, Runa Lazzarino, John Welch, Andrew Jonathan Brent, Anne Kinderlerer, Peter Ghazal, Anthony C Gordon, Shashank Patil, Graham Cooke, DiAlS Co-Investigators, Cerie Costelloe, Ceire E Costelloe

**Affiliations:** 1 Division of Clinical Studies, Institute of Cancer Research, Sutton, London, UK; 2 GBSH, University College London, London, UK; 3 Nuffield Department of Primary Care and Health Sciences, University of Oxford, Oxford, UK; 4 Critical Care Department, University College Hospital, London, UK; 5 Oxford University Hospitals NHS Foundation Trust, Oxford, UK; 6 Nuffield Department of Medicine, University of Oxford, Oxford, UK; 7 St Mary’s Hospital, London, UK; 8 Cardiff University, Cardiff, UK; 9 Imperial College London, London, UK; 10 Chelsea and Westminster NHS Foundation Trust, London, UK; 11 Department of Infectious Disease, School of Medicine, Imperial College, London, UK; 12 NIHR BRC of Imperial College NHS Trust, London, UK

**Keywords:** Electronic Health Records, Decision Support Systems, Clinical, Emergency Service, Hospital

## Abstract

**Introduction:**

The National Health Service (NHS) ‘move to digital’ incorporating electronic patient record systems (EPR) facilitates the translation of paper-based screening tools into digital systems, including digital sepsis alerts. We evaluated the impact of sepsis screening tools on in-patient 30-day mortality across four multi-hospital NHS Trusts, each using a different algorithm for early detection of sepsis.

**Methods:**

Using quasi-experimental methods, we investigated the impact of the screening tools. Individual-level EPR data for 718 000 patients between 2010 and 2020 were extracted to assess the impact on a target cohort and control cohort using interrupted time series analysis, based on a binomial regression model. We included one Trust which uses a paper-based screening tool to compare the impact of digital and paper-based interventions, and one Trust which did not introduce a sepsis screening tool, but did introduce an EPR.

**Results:**

All Trusts had lower odds of mortality, between 5% and 12%, after the introduction of the sepsis screening tool, before adjustment for pre-existing trends or patient casemix. After adjustment for existing trends, there was a significant reduction in mortality in two of the three Trusts which introduced sepsis screening tools. We also observed age-specific effects across Trusts.

**Conclusion:**

Our findings confirm that patients with similar profiles have a lower mortality risk, consistent with our previous work. This study, conducted across multiple NHS Trusts, suggests that alerts could be tailored to specific patient groups based on age-related effects. Different Trusts may require unique indicators, thresholds, actions and treatments. Including additional EPR information could further enhance personalised care.

**WHAT IS ALREADY KNOWN ON THIS TOPIC**

**WHAT THIS STUDY ADDS**

- This is the first evaluation of digital sepsis screening tools across multiple National Health Service hospitals in England. The results show that the implementation of these digital screening tools is associated with a reduction in mortality associated with sepsis. The results also show that there are differential effects of these tools in different age groups.

**HOW THIS STUDY MIGHT AFFECT RESEARCH, PRACTICE OR POLICY**

- We have shown that the picture is complex; adjusting for patient mix and pre-existing trends, the impact of sepsis screening tools differs for specific patient groups. Recent guidance from the UK National Institute for Clinical Excellence (NICE) calls for tailored approaches to screening for sepsis, and our findings support these calls. We propose that incorporating further information from the electronic patient record could facilitate tailoring of these digital tools for specific patient populations.

## Introduction

 Sepsis is an international public health problem. Screening for sepsis is widely implemented across countries as an essential approach to facilitate prompt treatment and improve patient outcomes in hospital settings, and protocols to support early identification and standardised treatment of sepsis have been developed all over the world, but vary in their approach.^1^ The National Health Service (NHS) in England has introduced various incentives to improve sepsis screening in UK hospitals.^2^ Many screening tools which are used to support early identification of patients with infection and at risk of developing organ failure were originally designed as diagnostic criteria.^3^ In England, all healthcare organisations are expected to use the National Early Warning Score (version 2) (NEWS2), a generic screening tool indicating deterioration and hence the possibility of sepsis. There is a clear advantage of a common language being used across all organisations. Inada-Kim^4^ warns against ‘blinkered condition-specific approaches’ particularly when patients are admitted as emergencies.

The NHS ‘move to digital’ through the incorporation of electronic patient record systems (EPR) facilitates the translation of paper-based sepsis screening tools into digital systems, including digital sepsis alerts (DSAs). To improve care for patients with sepsis, comply with national financial incentive programmes and make best use of the introduction of EPR, hospitals in England have introduced DSAs. A variety of algorithms are in use, with different workflows and different implementation strategies. A recent survey of NHS Trusts in England suggests that EPR systems have been adopted by 89% of trusts, an increase from 77% in 2018.^2^ It is not known whether the translation of traditional paper-based screening tools into digital systems improves patient outcomes.

Digital alerts have generally been introduced across hospitals without randomisation or in a phased approach which would have supported rigorous evaluation of their impact. A systematic review in 2019 found that the implementation of digital sepsis screening tools was linked to improved patient outcomes, including reductions in length of stay, but no evidence of associations with mortality or time to antibiotics.^5^ A London-based study in 2020 examined the impact of sepsis alerts introduced in a phased approach and was evaluated using inverse probability of treatment weighting, common in the analysis of natural experiments to emulate a randomised controlled trial (RCT) using real world healthcare data. This showed the introduction of DSAs was associated with a 23% lower risk of death within 30 days.^6^ Alturki *et al*^7^ found similar reductions in hospital mortality in children. It is not clear that current sepsis screening tools and treatments are equally effective in all patient groups. Some evidence suggests that sepsis is an end-of-life condition and rapid treatment may be more important for younger patients.^8^ Recently, a large study has shown that patients from deprived backgrounds are more likely to have sepsis and more likely to die from sepsis.^9^

In this multi-site study, we aimed to determine the impact of the introduction of sepsis screening tools on in-patient 30-day mortality. To determine whether any impact on patient outcomes is associated with the digital nature of some sepsis screening tools, we included a Trust which introduced a paper-based alert. To further understand whether changes in patient outcomes associated with the introduction of DSAs, we included a fourth Trust and considered the impact of the introduction of electronic health records as a sensitivity analysis. We considered whether screening tools have a different impact on younger patients or people from more deprived backgrounds.

## Methods

This was a retrospective study to analyse the impact of the introduction of different sepsis screening tools in three NHS Trusts, with a fourth Trust acting as a control. The interventions in each Trust are shown in table 1 and summarised by Lazzarino *et al*.^10^

**Table 1:** The interventions and key dates for four NHS Trusts included in the study

Trust	Sepsis screening tool		Date of introduction	Period of inclusion
**A**	Paper based	Based on ‘Red Flag Sepsis’*	April 2016	March 2010 to February 2020
**B**	Digital	Based on ‘Red Flag Sepsis’*, locally adapted. Calculated whenever clinical observations are entered into the EPR.	May 2016	February 2013 to February 2020
**C**	Digital	Alert packaged as part of Cerner’s EPR—the St John Sepsis Algorithm†. Calculated whenever clinical observations are entered into the EPR.	April 2017	April 2010 to February 2020
**D**	None—EPR introduction is intervention of interest		March 2019	April 2016 to February 2020

* Red flag sepsis includes clinical observations and lactate levels. See online supplemental material table 1Table 1 Supplementary materials in Kopcynska^25^ (2018)for further details.

† St John’s Sepsis Algorithm is based on clinical observations and blood test results. See Honeyford^6^ (2020) for further details.

EPR, electronic patient record; NHS, National Health Service.

### Timeline

Trusts provided data from as early as 1 April 2010; we included data from the first day of the month where data for the full month were available. The period of study ended on 31 January 2020 to account for the potential impact of COVID-19.

In order to use all available data, we have to account for different timings of the introductions of sepsis screening tools. We do not have identical periods of data for all Trusts.

The periods of study and the date of introduction of sepsis tools are shown in table 1.

### Study design and population

Data for all adult (18+) inpatients admitted between 1 April 2010 and 31 January 2020 were initially eligible for inclusion in the study.

We identified two cohorts of patients using ICD-10 codes in the patients’ record:

Suspicion of sepsis (SoS) cohort: Patients we expected to be impacted by the introduction of a sepsis screening tool. We used published ICD-10 codes associated with bacterial infections that can cause sepsis.^7^ This group is thought to mitigate against bias introduced through changes in coding practices. See box 1 for more details.

Box 1 **Identifying patients with sepsis using routinely collected, structured data**

A key challenge in evaluating interventions to improve outcomes for patients with sepsis is that neither case note review nor administrative records are necessarily reliable for identifying patients with sepsis. The heterogeneric nature of sepsis means diagnosing sepsis involves considerable subjectivity.^26^ Studies have found that relying on International Classification of Diseases version 10 (ICD-10) sepsis-specific codes in administrative data can lead to underestimates of sepsis incidence,^27^ particularly in patients with less severe sepsis.^28^ In addition, sudden changes in coding practice can have a big impact.^29^ Many interventions, which often target sepsis awareness, can also lead to increases in the recording of sepsis. Digital interventions, such as the ones studied in this paper, often automate the coding of sepsis based on clinician responses, so may also increase the recording of ICD-10 sepsis codes. An additional challenge, which is less discussed, involves the definition of sepsis as an infection which leads to organ dysfunction. Rhee *et al*^26^ have pointed out that it is not always clear whether the organ dysfunction is a result of the infection, but in addition, a strong sepsis intervention may reduce the opportunity of infection resulting in organ dysfunction, if the infection is identified and treated earlier. This may lead to a decrease in patients with sepsis but may not improve more severe outcomes such as mortality.

These many challenges mean that when studying interventions to improve outcomes for patients with sepsis, focusing only on patients with an ICD-10 code for sepsis is likely to be biased, and the cause of that bias is multifactorial and is likely to be influenced by the intervention.

In this study, we use an established list of infection codes to define the denominator. These codes have been shown to be more resistant to bias, and in this study, we include sensitivity analysis to determine if this is the case in the Trusts we have included in our analysis.

The list was developed by Inada-Kim *et al*^30^ and is known as the suspicion of sepsis code list; a list of codes which ‘identifies patients with a bacterial infection serious enough to warrant admission’^12^ and considered to be as inclusive as possible.

Control cohort: A comparator group of patients whose outcomes we did not expect to be impacted by the introduction of the sepsis screening tool. These patients had had an upper gastrointestinal bleed.^11^ We excluded patients who had an ICD-10 code included in the SoS list.

### Data

EPR data were provided by NHS Trusts. Data used in this study were routinely collected, processed by Trusts to comply with NHS requirements for secondary uses service.^12^ These data are quality checked by individual Trusts before being submitted to the NHS and are compiled into Hospital Episode Statistics which have been widely used for research in the UK.^13^ Data are stored by Trusts and were made accessible to us via secure data environments, with appropriate data sharing and access government arrangements. See box 2 for additional information.

Box 2 **Data sharing**

A key strength of this study was the collaboration between four NHS Trusts providing data in sharing, analysing and interpreting data. The NIHR-Health Informatics Collaborative facilitated data sharing agreements between the NHS Trusts. We worked with clinicians and health informatics specialists to develop a data dictionary enabling a wide range of research projects. Health informatics managers at each NHS trust quality checked and processed the data according to the data dictionary, before subsequent transfer to either Imperial Clinical Analytics, Research and Evaluation^31^ or Biomedical Research Informatics Digital Environment^32^ secure data environments.

NHS, National Health Service. NIHR, National Institute of Health Research

### Data processing

Each patient admission was treated as a separate event with a binary outcome: in-hospital mortality within 30 days of admission. Patient demographic information was linked to hospital admissions through unique patient IDs. Patients were excluded if their age or gender was missing or if their patient ID was missing. Less than 15 hospital admissions were excluded in each Trust.

### Statistical analysis

#### Main analysis

Descriptive statistics were used to summarise in the patient cohorts over time and between Trusts.

We assessed the impact of the introduction of DSAs, paper sepsis screening tools and the introduction of an EHR on in-hospital mortality in the SoS cohort and separately on the control cohort using an interrupted time series (ITS) study design, based on a segmented binomial regression model, including a time index and an intervention variable to indicate whether the time variable is before or after the intervention as independent variables.^14^ This design means trends before the intervention are included in the model, allowing us to compare the actual mortality rate after the intervention with the counterfactual, that is, the predicted mortality if there had been no intervention.^15^

To reduce potential bias introduced by differences in the preintervention and postintervention cohorts, we adjusted for patient casemix, including age, ethnicity, gender and comorbidities using the weighted Elixhauser score. We also adjusted for hour of admission and seasonality.

A priori*,* we considered whether sepsis alerts might have an impact on different patient groups; we hypothesised that sepsis screening tools might have different effects on older, frailer patients and patients with a higher level of comorbidities. We, therefore, modelled two interaction terms: age and comorbidities in separate models.

### Sensitivity analyses

Sepsis incidence: To confirm our hypothesis that studying patients coded as having sepsis can be impacted by coding practice, as described in box 1, we plotted the incidence and mortality in patients with an SoS code and a sepsis code over time.

Additional adjustment for deprivation: We modelled the impact of the introduction of a sepsis screening tool adjusted for deprivation for these Trusts for the two Trusts that were able to supply data on deprivation, based on the Index of Multiple Deprivation (IMD) score.^16^

## Results

In total, we examined mortality patterns in 607 980 SoS patients across three Trusts. In Trust A, we included 156 387 patients who were admitted over 119 months, in Trust B 248 301 patients over 85 months, in Trust C 203 212 over 118 months and Trust D 110 110 over 46 months.

In all Trusts, the SoS cohort had more females than males, with a high proportion aged 65 and over, and the majority exhibiting at least one comorbidity. Admission rates were lowest between 20:00 and 07:00. The admission rate was similar in the winter and non-winter months. Approximately 40% of SoS patients at Trusts A, C and D are coded as white British and Irish, compared with nearly 80% in Trust B. We included all patients, even those with missing ethnicity; ethnicity was not known (either not stated or missing) for approximately one-sixth of patients (see table 2 for more details).

**Table 2:** Summary of patients admitted with the SoS before and after the introduction of a sepsis screening tool

	Trust A*		Trust B†		Trust C†		Trust D‡	
	Before	After	Before	After	Before	After	Before	After
**No. of patients admitted**	79 527	76 860	66 419	181 882	116 404	86 888	43 046	67 064
**Gender**								
Male	34 495 (43.4)	33 709 (43.9)	31 257 (47.1)	86 356 (47.6)	52 033 (44.7)	39 002 (44.9)	19 903 (46.2)	32 130 (47.9)
Female	45 032 (56.6)	43 151 (56.1)	35 162 (52.9)	95 526 (52.4)	64 371 (55.3)	47 886 (55.1)	23 143 (53.8)	34 934 (52.1)
**Ethnicity**								
White British and Irish	34 409 (43.3)	31 053 (40.4)	53 296 (80.2)	136 944 (75.3)	51 324 (44.1)	30 006 (34.5)	19 081 (44.3)	27 490 (41)
Asian§	10 217 (12.8)	11 253 (14.6)	2313 (3.5)	6219 (3.4)	12 540 (10.8)	9298 (10.7)	3615 (8.4)	6342 (9.5)
Black¶	4160 (5.2)	3554 (4.6)	880 (1.3)	2462 (1.4)	13 354 (11.5)	9641 (11.1)	3401 (7.9)	6249 (9.3)
Any other ethnicity	17 108 (21.5)	16 582 (21.6)	2741 (4.1)	9998 (5.5)	27 813 (23.9)	21 707 (25.0)	8482 (19.7)	14 476 (21.6)
Not stated	9662 (12.5)	12 913 (16.8)	6920 (10.3)	24 861 (13.6)	5309 (4.6)	10 584 (12.2)	3791 (8.8)	11 171 (16.7)
Not known or missing	3971 (5.0)	1505 (2.0)	269 (0.4)	1443 (0.7)	6064 (5.2)	5652 (6.5)	4676 (10.9)	1336 (2)
Age**	49 (27, 64)	61 (37, 77)	55 (30, 70)	52 (29, 66)	55 (33, 70)	49 (27, 64)	59 (37, 76)	58 (36, 74)
Elixhauser score**	5 (0,12)	3 (0, 8)	3 (0, 8)	4 (0, 10)	4 (0, 10)	5 (0,12)	2 (0, 8)	4 (0, 11)
Season of admission								
Winter (Decemebr–March)	32 720 (37.7)	26 767 (34.8)	24 852 (37.4)	60 158 (33.1)	37 442 (32.2)	32 720 (37.7)	13 536 (31.4)	20 736 (30.9)
Spring, Summer, Autumn (April–November)	51 377 (62.3)	50 093 (65.2)	41 567 (62.6)	121 724 (66.9)	78 962 (67.8)	51 377 (62.3)	29 510 (68.6)	46 328 (69.1)
Time of admission								
Morning (7:00–9:00)	10 941 (12.6)	8182 (10.6)	9414 (14.2)	31 682 (17.4)	14 018 (12.0)	10 941 (12.6)	8227 (19.1)	13 768 (20.5)
Midday (10:00–13:00)	16 905 (19.5)	13 761 (17.9)	14 385 (21.7)	43 711 (24.0)	19 099 (16.4)	16 905 (19.5)	8646 (20.1)	14 217 (21.2)
Afternoon (14:00–19:00)	28 146 (32.4)	24 997 (32.5)	23 606 (35.5)	59 811 (32.9)	39 434 (33.9)	28 146 (32.4)	12 003 (27.9)	17 958 (26.8)
Night (20:00–6:00)	30 869 (35.6)	29 920 (38.9)	19 014 (28.6)	46 678 (25.7)	43 853 (37.7)	30 869 (35.6)	14 170 (32.9)	21 121 (31.5)
Patients who died	4200 (4.8)	3017 (3.9)	3979 (6.0)	9429 (5.2)	5834 (5.0)	4200 (4.8)	1205 (2.8)	2251 (3.4)

Counts and percentage of all admissions in brackets.

* Paper -based screening tool.

† Digital -based screening tool.

‡ EPR introduced.

§ Asian, Asian British and Mixed Asian .

¶ Black, Black British and Mmixed Bblack .

** Median and interquartile range IQR.

EPR, electronic patient record; SoS, suspicion of sepsis.

**Figure 1:**
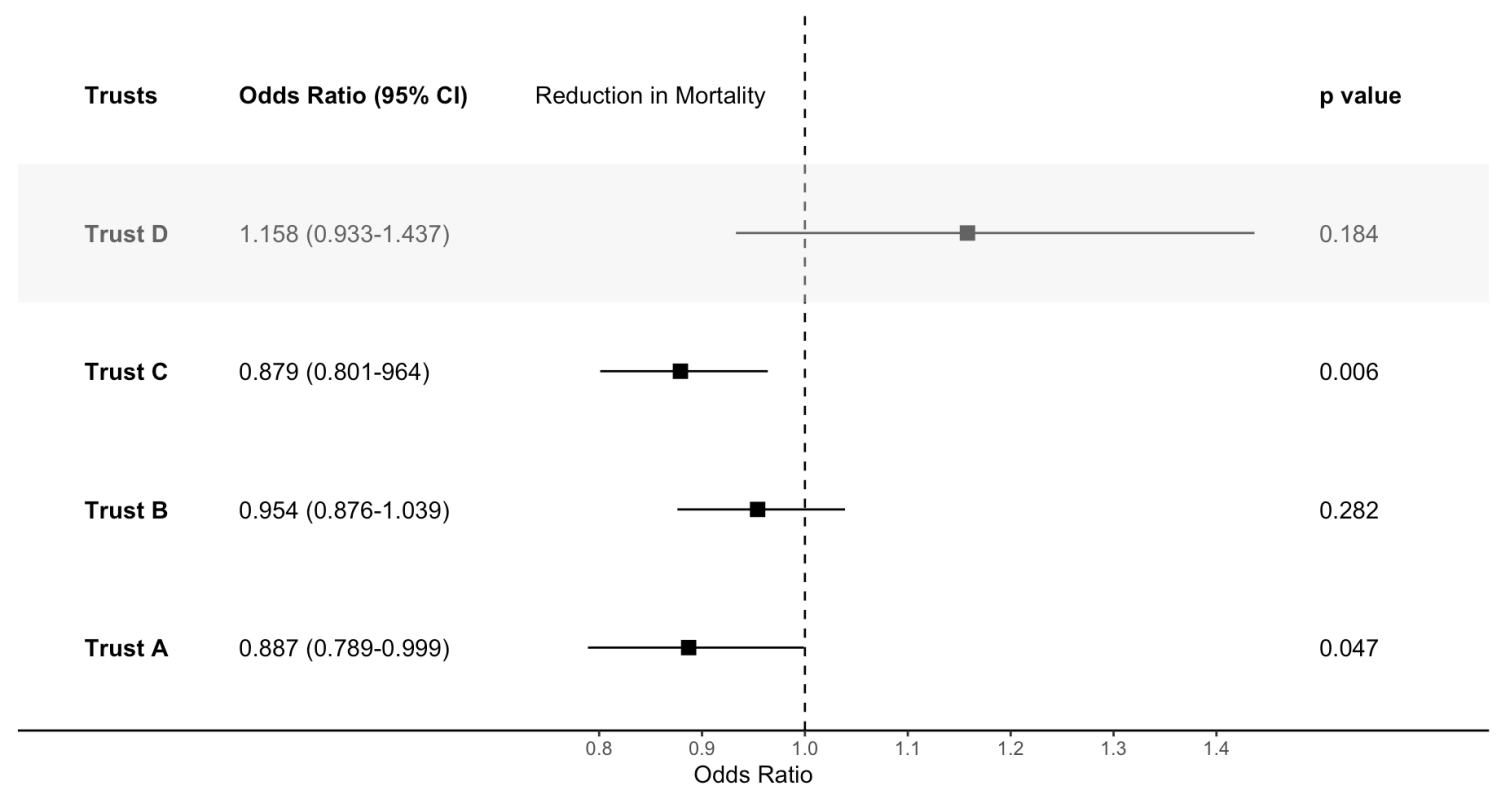
ORs for the impact of the introduction of a sepsis screening tool, adjusted for pre-existing trends, but not for casemix.

Trusts A, B and C had significantly lower mortality after the introduction of the sepsis screening tool before adjustment for pre-existing trends or patient casemix. After adjusting for pre-existing trends, there was a significant reduction in mortality in Trusts A and C, as shown in figures1  2.

**Figure 2:**
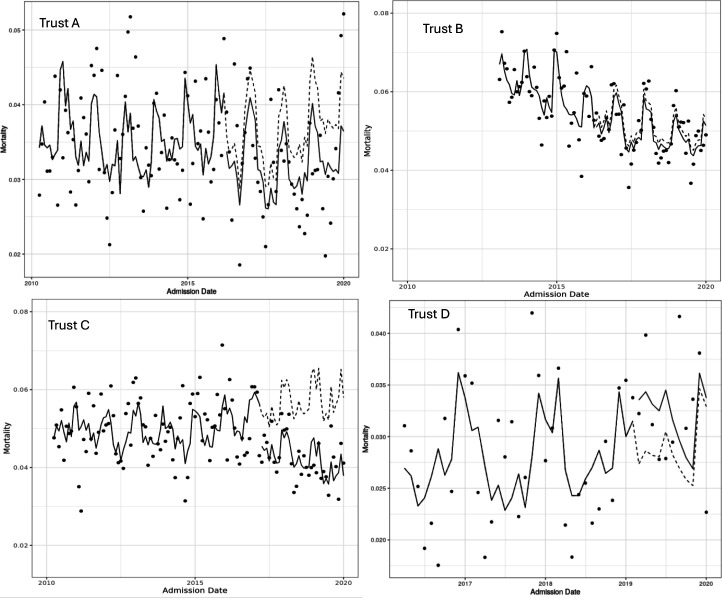
30-day mortality trend in the SoS cohort. Dots represent the actual mortality, the dashed line represents the counterfactual if there was no intervention and the solid line represents the modelled mortality pre the intervention, and post as if there was no intervention. SoS, suspicion of sepsis.

In Trust A, crude analysis indicated that there was a reduction in the mortality rate following the introduction of the screening tool (see figure 1). After adjusting for time and season of admission and patient casemix, the screening tool showed no impact on mortality (see table 3 for more details). We investigated whether the alert had differential impacts on specific patient groups by fitting interaction terms. This suggested that the introduction of the screening tool was significantly associated with a reduction in mortality in older patients, but not younger patients (see online supplemental table 2 for details). There was no evidence of a different impact on patients with more comorbidities.

**Table 3:** Interrupted time-series analysis of the impact of sepsis screening tools on mortality outcomes for SoS patients in three NHS Trusts in England

Variable	Trust A		Trust B		Trust C		Trust D	
	OR (95% CI)	P value	OR (95% CI)	P value	OR (95% CI)	P value	OR (95% CI)	P value
Alert/EPR introduction	0.931 (0.822, 1.054)	0.261	0.961 (0.877, 1.053)	0.391	0.826 (0.749, 0.911)	0.0001	1.271 (1.006, 1.605)	0.0442
Time in days since the start of the study period (gradient)	1.000 (1.000, 1.000)	0.003	1.000 (1.000, 1.000)	0.041	1.000 (1.000, 1.000)	0.0003	1.000 (1.000, 1.000)	0.1468
Time in years since the start of the study period	1.044 (1.020, 1.068)	0.003	0.963 (0.929, 0.998)	0.041	1.025 (1.012, 1.039)	0.0003	0.999 (0.998, 1.000)	0.2106
Change in gradient*	1.000 (1.000, 1.000)	0.133	1.000 (1.000, 1.000)	0.968	1.000 (1.000, 1.000)	<0.0001	1.227 (1.098, 1.372)	0.0003
Season of admission (reference is February–October) winter admission	1.174 (1.103, 1.250)	<0.0001	1.112 (1.061, 1.166)	<0.0001	1.097 (1.046, 1.151)	0.0001	1.000 (1.000, 1.000)	0.0000
Time of admission (reference is morning)								
Afternoon	1.526 (1.318, 1.767)	<0.0001	1.735 (1.583, 1.901)	<0.0001	1.446 (1.313, 1.593)	<0.0001	2.468 (2.001, 3.044)	<0.0001
Midday	1.380 (1.178, 1.618)	0.0001	1.630 (1.479, 1.796)	<0.0001	1.324 (1.190, 1.473)	<0.0001	1.402 (1.098, 1.791)	0.0068
Night	1.562 (1.351, 1.807)	<0.0001	1.856 (1.692, 2.037)	<0.0001	1.565 (1.422, 1.722)	<0.0001	2.837 (2.310, 3.485)	<0.0001
Ethnicity (reference is White British and Irish)								
Ethnicity—any other	0.658 (0.592, 0.732)	<0.0001	0.968 (0.832, 1.126)	0.675	1.042 (0.982, 1.106)	0.1771	0.815 (0.699, 0.952)	0.0096
Asian†	1.203 (1.096, 1.321)	0.0001	0.707 (0.578, 0.866)	0.0008	0.949 (0.875, 1.029)	0.2071	0.960 (0.782, 1.177)	0.6920
Black‡	0.550 (0.430, 0.703)	<0.0001	0.952 (0.712, 1.273)	0.741	0.820 (0.753, 0.894)	<0.0001	1.173 (0.955, 1.441)	0.1284
Not known or missing	1.011 (0.883, 1.157)	0.877	2.765 (2.064, 3.704)	<0.0001	0.905 (0.802, 1.023)	0.1103	1.589 (1.299, 1.944)	<0.0001
Not stated	0.878 (0.800, 0.964)	0.006	1.426 (1.325, 1.534)	<0.0001	1.309 (1.196, 1.433)	<0.0001	1.339 (1.109, 1.617)	0.0024
Age (in years)	1.051 (1.048, 1.053)	<0.0001	1.046 (1.044, 1.047)	<0.0001	1.040 (1.039, 1.042)	<0.0001	1.032 (1.029, 1.035)	<0.0001
Gender (male is reference): female	0.878 (0.826, 0.933)	<0.0001	0.892 (0.852, 0.934)	<0.0001	0.932 (0.890, 0.975)	0.0024	0.918 (0.828, 1.017)	0.1026
Elixhauser score	1.112 (1.108, 1.116)	<0.0001	1.114 (1.111, 1.118)	<0.0001	1.111 (1.108, 1.114)	<0.0001	1.116 (1.110, 1.123)	<0.0001

Interrupted time-series data are presented as odds ratioORs (95% CI CIs), except where specified. Values are given to three decimal places.

* Interaction between time in days since the start of the study period and the introduction of the alert.

† Asian, Asian British and Mmixed Asian.

‡ Black, Bblack British and Mmixed Bblack.

EPR, electronic patient record; NHS, National Health Service; SoS, suspicion of sepsis.

In Trust B, prior to the introduction of a DSA there was a decreasing trend in mortality in patients in the SoS cohort, and the association of the introduction of the alert with mortality is not significant in both crude and adjusted analyses. However, the interaction between age and the introduction of the alert is significant, suggesting that the alert had a significant impact on reducing mortality in older patients, but not in younger patients.

In Trust C, there was an increasing trend in mortality prior to the introduction of a DSA, and an increase in odds of mortality of 2.6% (95% CI 1.2% to 4.0%). The introduction of the alert is associated with a decrease in odds of mortality of 14% (95% CI 21% to 5%). In addition, after the introduction of the alert, the trend in mortality rate changes to a decreasing mortality rate. However, there was no significant interaction between the introduction of the alert and age, suggesting the alert does not have a differential impact in patients of different ages.

The introduction of the EPR in Trust D was apparently associated with increased odds of mortality of 27% (95% CI 0.6% to 60%). We propose that this is likely to be to do with coding and recording changes as a result of both the introduction of the EHR but also national sepsis coding guidelines in April 2017 and again April 2018, which have been shown to have centre-specific impacts on mortality.^17^

While the primary objective of the statistical models is to assess the impact of digital sepsis screening alerts following casemix adjustment, the significance of various casemix variables presents an intriguing and complex pattern. The risk of mortality rises with age over 18 and higher Elixhauser scores. Being female is associated with a decreased risk of death. Additionally, mortality risk increases when patients are admitted during winter and not in the morning. The influence and significance of ethnicity on the risk of mortality exhibit variations across trusts. For example, being Asian or Asian British was associated with 20% higher odds of death in Trust A (95% CI 10% to 32%), a 29% lower odds of death in Trust B (95% CI 13% to 42%) and no significant association with mortality in Trust C. In addition, night-time admissions had a higher risk of mortality, across all Trusts.

### Control cohort

To determine if any change in mortality rate was specific to patients with an infection rather than patients who are acutely deteriorating, we modelled mortality in patients with a gastric bleed, who did not also have an SoS diagnosis. There was no statistical evidence (p>0.05) that the introduction of sepsis screening tools was associated with a decrease in mortality in this cohort. Both increasing age and Elixhauser score were significantly associated with increased risk of mortality (see online supplemental tables 3,4 for more details).

### Sensitivity analyses

Sepsis incidence: We investigated whether our hypothesis that the introduction of DSAs and financial incentives associated with sepsis screening affected the number of people with a sepsis diagnosis, which would justify our approach of using patients with a diagnosis from the SoS code list, rather than patients with an ICD-10 code specifically for sepsis. We found that the incidence of sepsis increases when a sepsis screening tool is introduced, and again when the coding policy changed in England. Although the number of patients who died during these times also increases, the case fatality is less (see online supplemental figure 1).

### Additional adjustment for deprivation

Only two trusts provided data on deprivation. Including this in the model had no effect on the overall model interpretation. Patients with no deprivation recorded had the highest odds of death. For patients with a score, there was no significant association with mortality.

## Discussion

All Trusts had a lower mortality rate in patients with a serious infection, identified by the SoS code list, after the introduction of a sepsis screening tool. After adjusting for patient casemix, admission patterns and pre-existing trends, the introduction of a sepsis screening tool was significantly associated with a decrease in mortality rate in one Trust. In the remaining two Trusts, there was evidence that the introduction of a sepsis screening tool was associated with a reduction in mortality rate in older patients.

We have previously shown that patients for whom a DSA was active had a lower risk of mortality than for those who had a similar profile.^6^ These results confirm these results, across multiple NHS Trusts, this lower risk, even when previous trends are considered. Previously, paper-based screening tools have been shown to be associated with a reduced risk of mortality.^18^ We found evidence that the introduction of a paper-based screening tool impacted mortality in an older patient cohort only. We have not found previous research which has looked at the differential impact of the sepsis screening tools on different groups of patients. The literature on the effectiveness of digital sepsis screening tools shows that our findings, with different impact in different healthcare organisations, are consistent. Evidence is unclear, and the rationale for different impacts in different settings is not well understood. Trust C’s algorithm includes blood test results, which may be more useful, or perhaps the alert introduced at Trust C was the right alert for the patient population at the right time, and this is why a clear impact was seen. The underlying trend in mortality prior to introduction may also be important.

Crude changes in mortality rates may show that sepsis screening tools reduce mortality, and many NHS Trusts have highlighted the impact of new tools.^19^ We have shown that the picture is more complex, and after adjusting for patient mix and pre-existing trends, the impact of sepsis screening tools may not be clear.

Across England, digital screening tools in NHS hospitals are based on paper-based screening tools embedded in EPRs^2^; generally, they do not exploit the extensive data held in EPRs or use machine learning algorithms to personalise alerts. The picture with digital screening tools is complex. For example, evaluations of the Epic Sepsis Model have suggested a reduced risk of mortality,^20^ but some suggest possible harm due to its poor diagnostic performance.^21^ Digital screening tools embedded in EPRs have advantages when compared with paper-based screening systems. They can be linked directly to treatment plans, which has been shown to improve adherence.^22^

A recent national study suggested that people of white ethnicity had the highest sepsis mortality risk.^9^ We found different mortality risks for different ethnic groups between Trusts, suggesting that the impact of ethnicity is different in different NHS Trusts. We retained ‘not stated’ and ‘missing’ as different ethnic groups and saw different odds of mortality, suggesting different Trusts may use these codes differently. The groups in use may not best describe ethnic groups in different areas of England and may not be comparable internationally.^23^ We explored deprivation in two trusts and did not find poorer outcomes for patients from more deprived areas but did find having no IMD score was associated with poorer outcomes. This group may be more likely to have no permanent residence, and therefore, be representative of the most vulnerable patients.

As with many studies on sepsis, we are limited by the challenges of a gold standard for sepsis diagnosis. We used the diagnosis list suggested by Inada-Kim.^4^ We have explored this in box 1. There was an increasing trend in SoS admissions during the period of study. Possible causes for this include increasing numbers of admissions, or increasing admissions of patients with infections, or less severely ill patients being included in the cohort due to changes in coding. The use of an ITS approach takes into account underlying trends and helps to disentangle these from the impact of interventions. Despite using this recommended method for determining causal inference,^24^ we cannot determine that the sepsis screening tools are solely responsible for changing behaviour which leads to reductions in mortality, and other confounders such as changes in staffing, pressures in the hospital, paramedic responses to suspected sepsis and treatment plans may be important factors in the changes we observed. Another factor to consider is the potential overlap between patients with an SoS cohort and those who triggered an alert. A further, detailed patient-level analysis which considers this in detail is in progress and part of ongoing work for a separate publication. We carried out sensitivity analysis to determine if any changes in the mortality risk in SoS patients were also seen in patients with a non-related condition and found no evidence of an associated reduction in mortality risk. In addition, we have shown that the introduction of an EPR was not associated with reduced mortality. In the future, we will look at patient-level analysis

Screening tools and digital alerts should be designed with a strong evidence base. Different patient groups within different Trusts may need different indicators, different thresholds for action and/or different actions and treatments. A parallel qualitative study from our team^10^ highlights calls from healthcare practitioners, who advise that sepsis screening tools should be more specific, patient-based, target healthcare practitioner teams, be portable and remotely accessible, and integrate community, ambulance and primary care with secondary care to accelerate emergency department (ED) triaging. A key advantage of EPRs and embedded digital tools is that screening and treatment can be readily personalised, without expecting healthcare professionals to look up specific guidance. Our results also support recent UK NICE guidelines which highlight that current sepsis screening tools are dependent on use of individual variables informed by low quality evidence.

For evidence-based screening tools, we need to have strong evaluations of interventions in healthcare and work collaboratively to share data and research methodology. The rich data from EPRs are a vital resource for identifying the need for digital innovation; developing and validating models; and evaluating interventions. We have shown that with detailed preparation and effective collaboration, we can establish results which support a wider understanding of the complex nature of preventing mortality from sepsis.

## Supplementary material

10.1136/bmjhci-2024-101141 online supplemental file 1

## Data Availability

Data are available on reasonable request.

## References

[1] Alexiou A, Rau C (2023). BMJ Best Practice: Sepsis in adults.

[2] Honeyford K, Nwosu A-P, Lazzarino R (2023). Prevalence of electronic screening for sepsis in National Health Service acute hospitals in England. BMJ Health Care Inform.

[3] Svendsen M, Steindal SA, Hamilton Larsen M (2023). Comparison of the systematic Inflammatory response syndrome and the quick sequential organ failure assessment for prognostic accuracy in detecting sepsis in the emergency department: A systematic review. Int Emerg Nurs.

[4] Inada-Kim M (2022). NEWS2 and improving outcomes from sepsis. *Clin Med (Lond*).

[5] Joshi M, Ashrafian H, Arora S (2019). Digital Alerting and Outcomes in Patients With Sepsis: Systematic Review and Meta-Analysis. J Med Internet Res.

[6] Honeyford K, Cooke GS, Kinderlerer A (2020). Evaluating a digital sepsis alert in a London multisite hospital network: a natural experiment using electronic health record data. *J Am Med Inform Assoc*.

[7] Alturki A, Al-Eyadhy A, Alfayez A (2022). Impact of an electronic alert system for pediatric sepsis screening a tertiary hospital experience. Sci Rep.

[8] Maley JH, Worsham CM, Landon BE (2020). Association between Palliative Care and End-of-Life Resource Use for Older Adults Hospitalized with Septic Shock. *Annals ATS*.

[9] Zhong X, Ashiru-Oredope D, Pate A (2023). Clinical and health inequality risk factors for non-COVID-related sepsis during the global COVID-19 pandemic: a national case-control and cohort study. *eClinicalMedicine*.

[10] Lazzarino R, Borek AJ, Honeyford K (2024). Views and Uses of Sepsis Digital Alerts in National Health Service Trusts in England: Qualitative Study With Health Care Professionals. *JMIR Hum Factors*.

[11] Ahmed A, Armstrong M, Robertson I (2015). Upper gastrointestinal bleeding in Scotland 2000-2010: Improved outcomes but a significant weekend effect. World J Gastroenterol.

[12] NHS Digital (2023). Secondary Uses Service.

[13] Chaudhry Z, Mannan F, Gibson-White A (2017). Research Outputs of England’s Hospital Episode Statistics (HES) Database: Bibliometric Analysis. Jhi.

[14] Schaffer AL, Dobbins TA, Pearson SA (2021). Interrupted time series analysis using autoregressive integrated moving average (ARIMA) models: a guide for evaluating large-scale health interventions. *BMC Med Res Methodol*.

[15] Lopez Bernal J, Cummins S, Gasparrini A (2018). The use of controls in interrupted time series studies of public health interventions. Int J Epidemiol.

[16] Ministry of housing, communities and local government National Statistics English indices of deprivation 2019.

[17] Atkin C, Pankhurst T, McNulty D (2022). The impact of changes in coding on mortality reports using the example of sepsis. BMC Med Inform Decis Mak.

[18] Tedesco ER, Whiteman K, Heuston M (2017). Interprofessional Collaboration to Improve Sepsis Care and Survival Within a Tertiary Care Emergency Department. J Emerg Nurs.

[19] NHS England (2019). Hundreds of lives saved through new tech to spot sepsis. https://www.england.nhs.uk/2019/08/hundreds-of-lives-saved-through-new-tech-to-spot-sepsis/.

[20] Cull J, Brevetta R, Gerac J (2023). Epic Sepsis Model Inpatient Predictive Analytic Tool: A Validation Study. *Crit Care Explor*.

[21] Wong A, Otles E, Donnelly JP (2021). External Validation of a Widely Implemented Proprietary Sepsis Prediction Model in Hospitalized Patients. JAMA Intern Med.

[22] Liengsawangwong R, Kumar S, Ortiz RA (2021). Health Informatics Tool Toward Sepsis Screening Perspect Health Inf Manag. Perspect Health Inf Manag.

[23] Routen A, Akbari A, Banerjee A (2022). Strategies to record and use ethnicity information in routine health data. Nat Med.

[24] Craig P, Katikireddi SV, Leyland A (2017). Natural Experiments: An Overview of Methods, Approaches, and Contributions to Public Health Intervention Research. Annu Rev Public Health.

[25] Kopczynska M, Sharif B, Cleaver S (2018). Red-flag sepsis and SOFA identifies different patient population at risk of sepsis-related deaths on the general ward. Medicine (Baltimore).

[26] Rhee C, Jones TM (2019). Centers for Disease Control and Prevention (CDC) Prevention Epicenters Program. Prevalence, Underlying Causes, and Preventability of Sepsis-Associated Mortality in US Acute Care Hospitals. JAMA Netw Open.

[27] Liu B, Hadzi-Tosev M, Liu Y (2022). Accuracy of *International Classification of Diseases*, 10th Revision Codes for Identifying Sepsis: A Systematic Review and Meta-Analysis. *Crit Care Explor*.

[28] Schwarzkopf D, Rose N, Fleischmann-Struzek C (2024). Understanding the biases to sepsis surveillance and quality assurance caused by inaccurate coding in administrative health data. Infection.

[29] Bateson M, Marwick CA, Staines HJ (2023). Performance of bedside tools for predicting infection-related mortality and administrative data for sepsis surveillance: An observational cohort study. PLoS One.

[30] Inada-Kim M, Page B, Maqsood I (2017). Defining and measuring suspicion of sepsis: an analysis of routine data. BMJ Open.

[31] NIHR Imperial Biomedical Research Council (2024). Imperial Clinical Analytics, Research and Evaluation.

[32] BRIDgE (2020). Biomedical Research Centre for Cancer.

