# Supplementary material for: Digital innovation in healthcare: quantifying the impact of digital sepsis screening tools on patient outcomes—a multi-site natural experiment
Source: BMJ Health Care Inform. 2025 Apr 27;32(1):e101141. doi: 10.1136/bmjhci-2024-101141 (PMC12035476; doi:10.1136/bmjhci-2024-101141)
Supplement: online supplemental file 1 [file bmjhci-32-1-s001.pdf]

Supplementary Materials

Table of Contents

Supplementary Materials **1**
A ICD-10 Codes for Gastrointestinal Bleeding 1
B Model Results for SoS patients when interaction terms are included 2
C Control condition 3
D Sensitivity Analysis 5

A ICD-10 Codes for Gastrointestinal Bleeding

ICD-10 Code	ICD-10 Description
I850	Oesophageal varices with bleeding
K226	Gastro-oesophageal laceration - haemorrhage syndrome K228 Other specified diseases of oesophagus
K250	Gastric ulcer, acute with haemorrhage
K252	Gastric ulcer, acute with both haemorrhage and perforation
K254	Gastric ulcer, chronic or unspecified with haemorrhage K256 Chronic or unspecified Gastric ulcer with both haemorrage and perforation
K260	K260 Duodenal ulcer, acute with haemorrhage
K262	K262 Duodenal ulcer, acute with both haemorrhage and perforation
K264	K264 Duodenal ulcer, chronic or unspecified with haemorrhage K266 Chronic or unspecified Duodenal ulcer with both haemorrage and perforation
K270	K270 Peptic ulcer, acute with haemorrhage
K272	Peptic ulcer, acute with both haemorrhage and perforation
K274	Peptic ulcer, chronic or unspecified with haemorrhage
K276	Chronic or unspecified peptic ulcer with both haemorrhage and perforation
K280	Gastrojejunal ulcer, acute with haemorrhage
K282	Gastrojejunal ulcer, acute with both haemorrhage and perforation
K284	Gastrojejunal ulcer, chronic or unspecified with haemorrhage
K286	Chronic or unspecified Gastrojejunal ulcer with both haemorrhage and perforation
K290	Acute haemorrhagic gastritis
K920	Haematemesis
K921	Melaena
K922	Gastrointestinal haemorrhage, unspecified

Table S1: ICD10 codes for gastrointestinal bleeding

B Model Results for SoS patients when interaction terms are included

	Trust A (CW)		Trust B (Ox)		Trust C (IC)	
Variable	OR (95% CI)	*p* value	OR (95% CI)	*p* value	OR (95% CI)	*p* value
Alert introduction	1.479 (1.014, 2.157)	0.042	1.310 (1.026, 1.674)	0.0306	1.052 (0.824, 1.343)	0.685
Time in days since the start of the study period (gradient)	1.000 (1.000, 1.000)	0.0004	1.000 (1.000, 1.000)	0.0595	1.000 (1.000, 1.000)	0.0002
Time in **years** since the start of the study period	0.959 (0.937, 0.982)	0.0004	0.966 (0.931, 1.001)	<0.0001	1.026 (1.012, 1.040)	0.0002
Change in gradient[1]	1.000 (1.000, 1.000)	0.1037	1.000 (1.000, 1.000)	0.8641	1.000 (1.000, 1.000)	<0.0001
Season of admission (reference is Feb – Oct) Winter admission	1.174 (1.103, 1.250)	<0.0001	1.113 (1.061, 1.167)	<0.0001	1.097 (1.047, 1.151)	0.0001
Time of admission (reference is Morning)	1.523 (1.316, 1.763)	<0.0001	1.735 (1.583, 1.901)	<0.0001	1.446 (1.313, 1.593)	<0.0001
Afternoon						
Midday	1.377 (1.175, 1.614)	0.0001	1.632 (1.480, 1.798)	<0.0001	1.323 (1.189, 1.472)	<0.0001
Night	1.560 (1.349, 1.804)	<0.0001	1.856 (1.692, 2.037)	<0.0001	1.565 (1.423, 1.722)	<0.0001
Ethnicity (reference is White British and Irish)						
Ethnicity – Any other	0.659 (0.593, 0.733)	<0.0001	0.967 (0.831, 1.124)	0.6594	1.043 (0.982, 1.107)	0.1713
Asian[2]	1.204 (1.097, 1.322)	0.0001	0.709 (0.579, 0.868)	0.0009	0.950 (0.876, 1.030)	0.2152
Black[3]	0.550 (0.430, 0.703)	<0.0001	0.952 (0.712, 1.273)	0.7289	0.822 (0.755, 0.895)	<0.0001
Not Known or Missing	1.011 (0.883, 1.157)	0.8785	2.759 (2.060, 3.697)	<0.0001	0.905 (0.801, 1.022)	0.108
Not Stated	0.879 (0.801, 0.965)	0.007	1.425 (1.324, 1.533)	<0.0001	1.306 (1.193, 1.430)	<0.0001
Age (in years)	0.877 (0.825, 0.932)	<0.0001	0.892 (0.852, 0.934)	<0.0001	0.932 (0.890, 0.975)	0.0025
Gender (male is reference) Female	1.053 (1.050, 1.056)	<0.0001	1.048 (1.046, 1.051)	<0.0001	1.041 (1.039, 1.043)	<0.0001
Elixhauser score	1.110 (1.105, 1.116)	<0.0001	1.114 (1.109, 1.119)	<0.0001	1.112 (1.109, 1.116)	<0.0001
Interaction age*alert	0.994 (0.989, 0.998)	0.0062	0.995 (0.992, 0.999)	0.0047	0.997 (0.994, 1.001)	0.1122
Interaction Elixhauser*alert	1.003 (0.996, 1.011)	0.3783	1.001 (0.995, 1.007)	0.6869	0.995 (0.990, 1.001)	0.0954

Table S2 Results for model with interaction terms fitted

[1]Interaction between time in days since the start of the study period and the introduction of the alert

C Control condition

Descriptive statistics for Trusts A to C for patients identified as having gastrointestinal bleeding,

	Trust A		Trust B		Trust C	
	Before	After	Before	After	Before	After
No. of admissions	4848	6300	3107	8406	5913	3661
Male	2666 (55)	3389 (53.8)	1569 (50.5)	4245 (50.5)	3489 (59)	2109 (57.6)
Female	2182 (45)	2911 (46.2)	1538 (49.5)	4161 (49.5)	2424 (41)	1552 (42.4)
White British & Irish	2109 (43.5)	2199 (34.9)	2684 (86.4)	6918 (82.3)	2631 (44.5)	1307 (35.7)
Asian[1]	596 (12.3)	989 (15.7)	99 (3.2)	235 (2.8)	5499 (93)	359 (9.8)
Black[2]	247 (5.1)	315 (5)	43 (1.4)	126 (1.5)	757 (12.8)	498 (13.6)
Any other Ethnicity	1004 (20.7)	1342 (21.3)	106 (3.4)	412 (4.9)	1360 (23)	901 (24.6)
Not Stated	674 (13.9)	1367 (21.7)	177 (5.7)	706 (8.4)	2424 (41)	326 (8.9)
Not Known or Missing	218 (4.5)	95 (1.5)	0 (0)	0 (0)	3725 (63)	267 (7.3)
Age[3]	55 (39, 71)	54 (39, 71)	59 (42, 71)	53 (36, 66)	54 (38, 68)	49 (34, 64)
Elixhauser score[3]	0 (0, 4)	0 (0, 5)	2 (0, 6)	3 (0, 9)	1 (0, 8)	5 (0, 12)
Season of admission - Winter	1643 (33.9)	2230 (35.4)	1081 (34.8)	2715 (32.3)	1821 (30.8)	1333 (36.4)
Spring, summer, autumn	3205 (66.1)	4070 (64.6)	2026 (65.2)	5691 (67.7)	4092 (69.2)	2328 (63.6)
Morning (7-9)	800 (16.5)	1172 (18.6)	587 (18.9)	1858 (22.1)	769 (13)	498 (13.6)
Midday (10-13)	1328 (27.4)	1821 (28.9)	994 (32)	2614 (31.1)	1177 (19.9)	805 (22)
Afternoon (14-19)	1585 (32.7)	2054 (32.6)	985 (31.7)	2581 (30.7)	2046 (34.6)	1252 (34.2)
Night (20-6)	1134 (23.4)	1260 (20)	541 (17.4)	1362 (16.2)	1922 (32.5)	1106 (30.2)
Mortality	53 (1.1)	82 (1.3)	65 (2.1)	252 (3)	154 (2.6)	73 (2)

Table S 3: Summary of patients admitted with the control condition (gastrointestinal bleeding - see Table S1)before and after the introduction of a sepsis screening tool. Counts and % of all admissions in brackets

[1]Asian, Asian British and Mixed Asian
[2]Black, Black British and Mixed Black
[3]Median and interquartile range

	Trust A		Trust B		Trust C	
Variable	OR (95% CI)	*p* value	OR (95% CI)	*p* value	OR (95% CI)	*p* value
Alert introduction	1.810 (0.773, 4.239)	0.171	1.139 (0.581, 2.233)	0.705	0.751 (0.378, 1.490)	0.413
Time in **days** from alert introduction (gradient)	1.000 (0.999, 1.000)	0.056	1.000 (0.999, 1.001)	0.631	1.000 (1.000, 1.000)	0.247
Time in **years** from alert introduction (gradient)	0.847 (0.714, 1.004)	0.056	0.935 (0.711, 1.230)	0.631	0.954 (0.880, 1.033)	0.247
Change in gradient[1]	0.999 (0.998, 1.000)	0.207	1.000 (0.999, 1.001)	0.903	1.000 (0.999, 1.001)	0.592
Season of admission (reference is Feb – Oct) Winter admission	0.710 (0.435, 1.159)	0.170	1.184 (0.836, 1.678)	0.341	1.155 (0.848, 1.573)	0.3622
Time of admission (ref is morning)	NA		NA	NA	NA	NA

Afternoon	1.221 (0.528, 2.824)	0.641	2.359 (1.233, 4.513)	0.010	1.478 (0.803, 2.720)	0.209
Midday	1.100 (0.444, 2.722)	0.837	1.495 (0.750, 2.982)	0.253	1.113 (0.562, 2.204)	0.759
Night	1.133 (0.482, 2.666)	0.775	2.927 (1.496, 5.725)	0.002	2.339 (1.290, 4.242)	0.005
Ethnicity (reference is White British and Irish)						
Ethnicity – Any other	0.615 (0.289, 1.310)	0.207	0.237 (0.031, 1.841)	0.169	0.974 (0.674, 1.407)	0.887
Asian[2]	1.326 (0.726, 2.420)	0.358	1.389 (0.411, 4.690)	0.597	1.053 (0.646, 1.718)	0.835
Black[3]	0.613 (0.169, 2.223)	0.457	0.626 (0.082, 4.777)	0.651	0.572 (0.333, 0.982)	0.0428
Not Known or Missing	3.094 (1.417, 6.753)	0.005	NA	NA	0.792 (0.375, 1.673)	0.541
Not Stated	0.619 (0.288, 1.334)	0.221	2.748 (1.648, 4.583)	<0.0001	0.978 (0.479, 1.998)	0.951
Age (in years)	1.030 (1.016, 1.044)	<0.0001	1.029 (1.017, 1.041)	<0.0001	1.019 (1.010, 1.029)	<0.0001
Gender (male is reference) Female	1.280 (0.820, 2.000)	0.277	1.045 (0.748, 1.461)	0.796	1.264 (0.940, 1.700)	0.120
Elixhauser score	1.201 (1.170, 1.233)	<0.0001	1.182 (1.157, 1.207)	<0.0001	1.138 (1.120, 1.157)	<0.0001

Table S4: Interrupted time-series analysis of the impact of sepsis screening tools on mortality outcomes for control patients in three NHS Trusts in England.

Interrupted time-series data are presented as odds ratios (95% CI), except where specified. Values are given to three decimal places

[1]*Interaction between time in days since the start of the study period and the introduction of the alert*

[2]*Asian, Asian British and Mixed Asian*

[3]*Black, Black British and Mixed Black*

D Sensitivity Analysis

We investigated the incidence and case mortality for the SoS and sepsis cohorts. These are shown below for two Trusts.

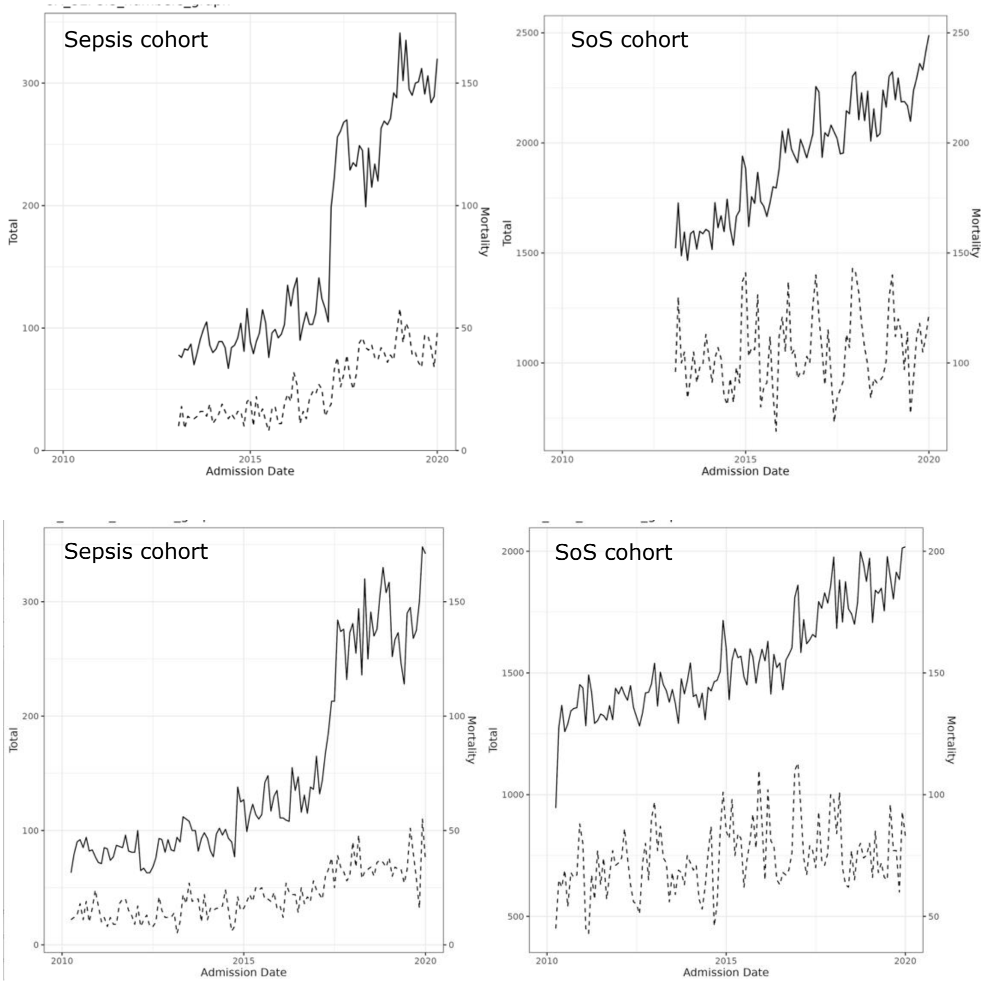

Figure S1: Incidence and mortality for the sepsis and SoS cohorts for two Trusts.

Dashed line shows number of deaths.
Solid line number of patients.
